# Reduction of posterior dislocated hip prosthesis using a modified lateral position maneuver: a retrospective, clinical comparative, and follow-up study

**DOI:** 10.1186/s12891-022-05876-8

**Published:** 2022-10-20

**Authors:** Gang Wang, Haoyang Wang, Jing Yang, Bin Shen, Zongke Zhou, Yi Zeng

**Affiliations:** Department of Orthopedics, Orthopedic Research Institute, West China Hospital, Sichuan University, Chengdu, 610041 Sichuan Province China

**Keywords:** Allis maneuver, Complications, Posterior dislocation, Reduction maneuver, Total hip arthroplasty

## Abstract

**Background:**

Posterior dislocation (PD) is a common complication after total hip arthroplasty (THA), and the Allis maneuver is the most widely used method for reduction. However, this classic maneuver has some disadvantages. The aim of the present study was to investigate whether a modified lateral position (MLP) reduction maneuver provides an easier and safer method for PD reduction than the Allis maneuver.

**Methods:**

Between August 2019 and September 2021, a series of 88 consecutive PD patients who underwent THA were retrospectively evaluated. The patients were divided into the MLP reduction group and Allis reduction group according to the electronic health medical record. The success rate of closed reduction, Harris hip score (HHS), and radiographic outcomes were determined. Satisfaction scores, doctor safety events and complications were also determined and compared between the groups. The mean follow-up period was 1.66 ± 0.88 years.

**Results:**

The success rate of reduction in the MLP group was significantly 12.5% higher than that in the Allis group *(P* = *0.024*). Periprosthetic fracture and implant loosening were retrospectively identified in 2 hips and 1 hip, which all occurred in the Allis group. The mean doctor and patient SAPS scores in the MLP group were 84.00 points and 76.97 points, respectively, which were significantly higher than those in the Allis group (72.12, *P* = 0.008 and 63.28 points, *P* = *0.001*). Four adverse events were reported in the Allis group, compared with 0 in the MLP group.

**Conclusions:**

For PD after THA, the MLP reduction maneuver can effectively increase the reduction success rate, satisfaction, and doctor safety without increasing the risk of complications compared with the traditional Allis supine reduction maneuver.

**Trial registration:**

This study was registered in the Chinese Clinical Trial Registry (ChiCTR2100054562) in December 19th 2021.

## Background

Total hip arthroplasty (THA) is the gold standard for the treatment of end-stage hip diseases, which can relieve patients' hip pain and improve their quality of life [1, 2]. THA is also an ideal treatment after the failure of conservative treatment and is used as a solution for hip fracture after internal fixation failure [3]. Although THA ranks among the more successful surgical procedures in the field of orthopedics, serious postoperative complications are still inevitable, including aseptic loosening, dislocation, and infection [4]. Dislocation is a common complication following THA [4, 5]. It has been reported that the dislocation rate after primary THA ranges from 0.2% ~ 10% per year, while the dislocation rate of patients after revision can be tenfold higher than that of primary THA [5, 6]. Meanwhile, in the international literature and registers, revision due to dislocation accounts for 9%-17.7% of all revisions of primary THAs, and it is therefore considered to be the second most common reason requiring revision after aseptic loosening following THA [5, 7].

Posterior dislocation (PD) is the major type of dislocation after THA [8, 9]. Generally, closed reduction is the first step of treatment for patients with PD. This procedure usually occurs in the emergency department (ED) with sedation and analgesia. The closed reduction maneuver was first described by Bigelow in 1870 [10–12]. Since then, multiple reduction maneuvers have been proposed, such as the Allis maneuver, Lefkowitz maneuver, and Captain Morgan maneuver [13]. Among them, the Allis maneuver is the most widely used maneuver for closed reduction in clinical practice [12]. This maneuver requires the application of longitudinal traction force along the length of the thigh with the patient in the supine position and additional pressure on the iliac crests to hold the pelvis down during the procedure. However, the disadvantages of the Allis maneuver are obvious [13, 14]: (1) the traction direction is opposite to the gravity of the patient’s thigh, and the doctor needs to provide a strong traction force; (2) the doctor faces the risk of low back injury who generally stands over the patient to pull up on the bent knee and significant force upon the back during the procedure; (3) the potential risk for a fall from a height because this maneuver requires the doctor to enter the bed with the patient, especially if the patient remains on a stretcher in the ED; and (4) if the traction is not adequate and in place, external rotation during the procedure may lead to complications, such as periprosthetic fracture and implant loosening.

We carried out this retrospective, clinical comparative study to introduce a modified lateral position (MLP) reduction maneuver for PD after THA. Compared with the classic Allis maneuver, this maneuver did not need to provide a large traction force and was safe for the doctor without entering the bed with the patient. Our hypothesis was that this modified maneuver might provide an easier and safer method for reduction than the Allis maneuver in terms of reduction success rate, complication incidence, patient and doctor satisfaction, and doctor safety.

## Materials and methods

This study was approved by the Clinical Trials and Biomedical Ethics Committee of West China Hospital (No. 2021[1329]) and registered in the Chinese Clinical Trial Registry in 19th December, 2021(ChiCTR2100054562).

### Study design

This is a retrospective study. Between August 2019 and September 2021, a series of consecutive adult patients presenting to our hospital with an isolated, unilateral dislocation of a prosthetic hip was considered for enrollment in this study. The patients were divided into the MLP reduction group and Allis reduction group according to the electronic health medical record. Reduction success rate, complication incidence, patient and doctor satisfaction, and doctor safety were compared between two group.

### Reduction procedures

**Fig. 1:**
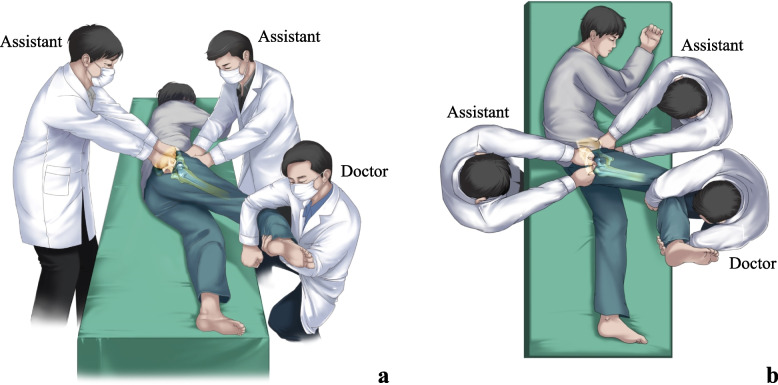
The diagram of the MLP maneuver. **a**. Front view of MLP reduction. **b**. The overhead view of MLP reduction

Before reduction, all the patients were examined fully and specifically for any neurovascular deficit of the affected leg. An anteroposterior X-ray of the pelvis was examined to confirm the diagnosis of PD. Patients were divided into the MLP reduction group and the Allis reduction group according to the reduction maneuver used:

1. MLP reduction group: a modified lateral position reduction maneuver originated from Skoff et al. [15]. The patient was placed in the lateral position on the stretcher or ED room bed with the affected limb facing up. One assistant applied a lateral force on the anterior superior iliac spine for pelvis stabilization and countertraction. The doctor stood in front of the patient and put the affected leg into 60 degrees of hip flexion, 40–45 degrees of internal rotation, and 40–45 degrees of adduction and flexes the knee to 90 degrees. The doctor applied a lateral and downward traction force mainly by using body weight without the back muscle involved. Another assistant stood behind the patient, palpated the protrusion in the gluteal region, and pushed the femoral head forward. The axial traction force and femoral head push force were applied to the distal and proximal femur, respectively, and gradually increased while the femur was slowly externally rotated until hip reduction succeeded (Fig. 1a-b).
2. Allis reduction group: the patient was placed in the supine position. The doctor entered the bed, stood above the patient, grasped the ipsilateral leg at the knee, and flexed the knee and hip to 90 degrees. An assistant stabilized the pelvis by firmly holding the pelvis down through pressure to both anterior iliac crests. The doctor applied axial traction to the distal femur and gradually externally rotated the femur to allow the artificial femoral head to enter the acetabular cup. Reduction was successful if an audible sound was heard.

The first attempt to reduce the dislocated hip was in the ED before admission. Before reduction, diazepam and dolantin were applied for sedation and analgesia. If failure occurred, the patient was admitted to the operating room, and closed reduction was attempted for a second time under the combined application of sedative anesthesia and muscle relaxants. If the second reduction still failed or complications occurred during reduction, such as fracture or implant loosening, open reduction was performed under general anesthesia.

After the reduction was finished, the patient was asked to undergo a check X-ray examination to confirm the success of the reduction. Following successful reduction, the hips were abducted, and a trapezoid-shaped pillow was placed between them for 3 months. Patients with multiple (≥ 2 times) dislocations were immobilized with a hip cast for 4–6 weeks, following a trapezoidal-shaped pillow for 3 months.

### Outcome assessment

The Harris Hip Score (HHS) [16] was measured to evaluate hip function and quality of life at the end of the follow-up period. At follow-up examinations, patients were asked if any other problems occurred after the operation, such as dislocation recurrence and hip dysfunction.

The radiographic analysis consisted of anteroposterior views of the pelvis and the hip and a true lateral view of the hip. The inclination angle of the acetabular cup component was determined by the method of Pradhan et al. [17]. Dislocation height was determined by the method of Sun et al. [18], which included perpendicular distances from the femoral head/neck junction and the tip of the greater trochanter to the interteardrop line.

The success rate of the first and the second closed reduction was determined and compared between the MLP group and Allis group. Complications were recorded for each patient in detail, such as periprosthetic fracture, implant loosening, and nerve injury.

The Self-Administered Patient Satisfaction Scale (SAPS) was applied to determine patient and doctor satisfaction [19]. A result was considered excellent if the SAPS score was between 75 and 100, good if between 50 and 74, fair if between 25 and 49, and poor if less than 25. In addition, doctor safety events were also determined, including doctors falling from the patient bed, lumbar muscle strain, upper limb muscle strain, etc.

### Statistical analysis

The distribution of baseline data and study outcomes were assessed with summary statistics, including means and standard deviations for quantitative data and frequencies and percentages for qualitative data. The symmetric distribution of continuous variables was compared between groups using the independent-samples t test, and categorical variables were compared using the chi-square test. For asymmetric variables, the Mann Whitney test was used among the two groups. The statistical analysis was performed using SPSS software (version 23.0; SPSS, Chicago, Illinois), with a *P* value < 0.05 considered significant.

## Results

**Fig. 2:**
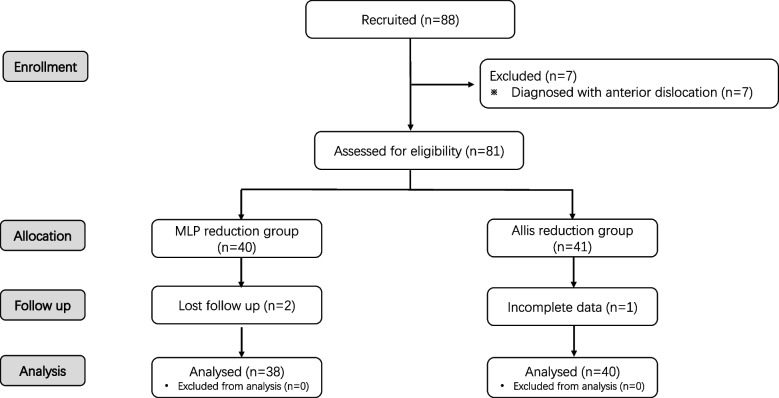
The flow diagram of patient enrollment. A total of 88 patients were recruited, and 81 were assessed for eligibility. Eventually, data from 78 patients were collected for statistical analysis

A total of 88 patients were enrolled in the study between August 2019 and September 2021, of which 7 were excluded because the diagnosis was anterior dislocation. Of the remaining 81 patients, 2 patients were lost to follow-up, and 1 patient had incomplete data. Finally, a total of 78 THA patients (78 hips) were confirmed to have PD and were enrolled for analysis (Fig. 2).

Patient demographic details are presented in Table 1. Sixty-nine patients were diagnosed with PD after primary THA, and 9 were diagnosed after revision. No significant difference was found in mean age, BMI, preoperative ROM (range of motor), comorbidities, or preoperative HHS for the two groups. The mean duration of follow-up was 1.66 ± 0.88 years.

**Table 1:** Baseline characteristics of the study population

Variable	MLP reduction group (*N* = 38)	Allis reduction group (*N* = 40)	*P* value*
Demographic characteristics			
Age (yr)	63.82 ± 14.91	58.72 ± 9.65	0.080
Male/female	19/19	19/21	0.825
Weight (kg)	64.65 ± 11.41	61.45 ± 11.85	0.227
Height (cm)	164.45 ± 8.15	161.63 ± 8.69	0.086
BMI (kg/m^2^)	24.12 ± 3.47	23.87 ± 3.82	0.762
Left/right/bilateral	8/21/9	8/19/13	0.690
Diagnosis			
Primary THA			
ONFH	19	18	0.658
OA	4	2	0.360
RA	3	5	0.503
DDH	8	10	0.903
Revision THA			
Prosthetic Loosening	2	2	0.958
Prosthetic Fracture	2	1	0.526
Infection	0	2	0.163
Comorbidities			
Hypertension	14	16	0.774
Diabetes mellitus	4	3	0.640
Osteoporosis	10	5	0.122
COPD	4	3	0.640
Preoperative hip function			
Flexion (deg)	90.26 ± 29.15	93.50 ± 31.07	0.637
Abduction (deg)	23.03 ± 10.81	24.75 ± 11.43	0.496
HHS score	28.42 ± 14.55	36.53 ± 15.65	0.582
Duration of follow-up (yr)	1.73 ± 1.94	1.60 ± 0.836	0.546

Abbreviations: *ONFH* osteonecrosis of the femoral head, *OA* osteoarthritis, *RA* rheumatoid arthritis, *DDH* developmental dysplasia of the hip, *COPD* chronic obstructive pulmonary disease, *HHS* Harris hip score

^*^The *p* value represents the result of independent-samples T test for continuous variables or the chi-square test for independent proportions that included the two groups

Table 2 shows the implant information and radiographic results of the two groups. No significant difference was found between the two groups in terms of acetabular cup size, femoral head size, femoral neck length, femoral stem type, or friction interface (*P* > 0.05). The mean acetabular cup inclination angles in the MLP and Allis groups were 38.66° and 38.64°, respectively, with no significant intergroup difference (*P* = 0.985). The mean dislocation height was 4.67 mm and 4.26 mm for the MLP and Allis groups, respectively, with no significant intergroup difference (*P* = 0.217).

**Table 2:** Implant information and radiographic evaluation

Variable	MLP reduction group (*N* = 38)	Allis reduction group (*N* = 40)	*P* value*
Implant information			
Acetabular cup size (mm)	50.68 ± 4.11	49.40 ± 4.01	0.167
Femoral head size (mm)	32.80 ± 3.09	32.13 ± 4.28	0.428
Length of femoral neck (mm)	1.91 ± 2.01	2.00 ± 1.69	0.827
Type of femoral stem			
Corail	30	28	0.366
Solution	3	3	0.948
S-Rom	2	3	0.687
Tri-lock	1	3	0.330
Summit	2	1	0.526
Spacer	0	2	0.163
Friction interface			
Ceramic on ceramic	31	32	0.860
Ceramic on polyethylene	7	6	0.685
Radiographic evaluation			
Inclination Angle of Cup(°)	38.66 ± 5.39	38.64 ± 5.75	0.985
Dislocation height (cm)	4.67 ± 1.23	4.26 ± 1.58	0.217

^*^The *p* value represents the result of independent-samples T test for continuous variables or the chi-square test for independent proportions that included the two groups

Table 3 shows the reduction results of the two groups. The MLP group had a significantly higher success rate of closed reduction than the Allis group (100% vs 87.5%, *P* = 0.024). The number of open reductions in the MLP group and Allis group was 0 and 5, respectively, with a significant difference between the groups (*P* = 0.024).

**Table 3:** Reduction results of the two groups

Variable	MLP reduction group (*N* = 38)	Allis reduction group (*N* = 40)	*P* value*
Reduction results			
Success rate of closed reduction (%)	38 (100%)	35 (87.5%)	**0.024**
The first reduction in ED (%)	33 (86.84%)	25 (62.50%)	**0.014**
The second reduction after admission (%)	5 (13.16%)	10 (25%)	0.185
No. of open reduction (%)	0 (0%)	5 (12.5%)	**0.024**
HHS score	88.50 ± 4.47	87.43 ± 8.36	0.479
Reduction complications			
Rate of complications (%)	0 (0%)	3 (7.50%)	0.085
Periprosthetic fracture (%)	0 (0%)	2 (5.00%)	0.163
Temporary spacer loosening (%)	0 (0%)	1 (2.50%)	0.327
Implants loosening (%)	0 (0%)	0 (0%)	–
Doctor Satisfaction			
SAPS score	84.00 ± 15.02	72.12 ± 22.92	**0.008**
Excellent (%)	31 (81.58%)	22 (55.00%)	**0.012**
Good (%)	5 (13.16%)	4 (10.00%)	0.663
Fair (%)	0 (0%)	5 (12.50%)	**0.024**
Poor (%)	2 (5.21%)	9 (22.50%)	**0.029**
Patient Satisfaction			
SAPS score	76.97 ± 15.79	63.28 ± 20.57	**0.001**
Excellent (%)	16 (42.11%)	6 (15.00%)	**0.008**
Good (%)	14 (36.84%)	18 (45.00%)	0.464
Fair (%)	8 (21.05%)	16 (40.00%)	0.070
Poor (%)	0 (0%)	0 (0%)	–

Abbreviations: *ED* emergency department, *HHS* Harris hip score, *SAPS* Self-Administered Patient Satisfaction Scale

^*^The *P* value represents the result of independent-samples T test for continuous variables or the chi-square test for independent proportions that included the two groups. *P* values with statistical significance are in bold print

**Fig. 3:**
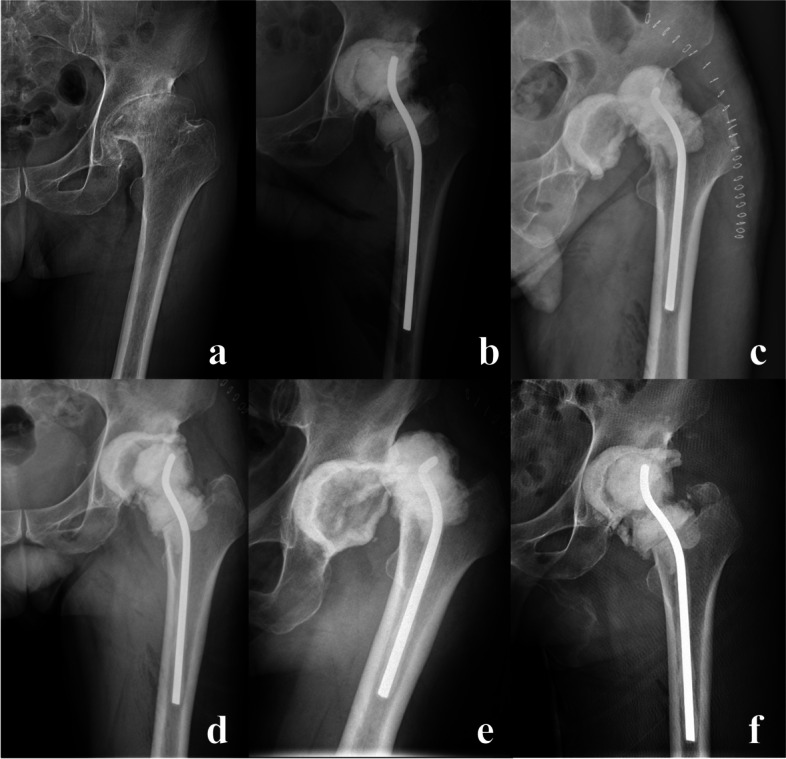
One case of periprosthetic fracture. A 67-year-old male patient was diagnosed with a purulent infection of the left hip and underwent debridement and antibiotic-loaded bone cement temporary spacers. PD occurred twice, and closed reduction was successful with the Allis maneuver. However, periprosthetic fracture of the greater trochanter was confirmed after the second reduction and treated with a conservative strategy because the spacers were stable. **a**. Preoperative X-ray film of the affected hip. **b**. Anteroposterior (AP) X-ray film of the affected hip immediately after revision. **c**. AP view X-ray film of the affected hip immediately after the first PD. **d**. AP view X-ray film immediately after the first reduction. **e**. AP view X-ray film immediately after the second PD. **f**. AP view X-ray film after the second reduction, showing greater trochanter fracture

**Fig. 4:**
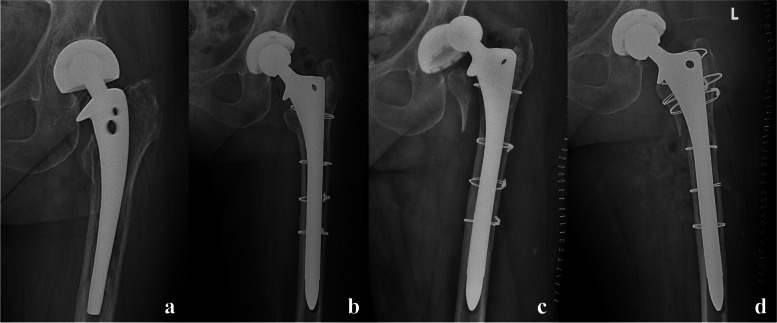
Another case of periprosthetic fracture. An 86-year-old female patient who developed severe osteolysis secondary to left hemiarthroplasty for femoral neck fracture 28 years ago and underwent total hip revision. PD was diagnosed 2 weeks after the operation, and a periprosthetic fracture occurred during the closed reduction process with the Allis maneuver. The prostheses were stable. Open reduction and internal fixation operations were performed under general anesthesia, and fracture fragments were fixed with wires. **a**. Preoperative X-ray film of the affected hip. **b**. AP view X-ray film of the affected hip immediately after revision. **c**. AP view X-ray film of the affected hip immediately after closed reduction with the Allis maneuver. **d**. AP view X-ray film of the affected hip immediately after open reduction and internal fixation

**Fig. 5:**
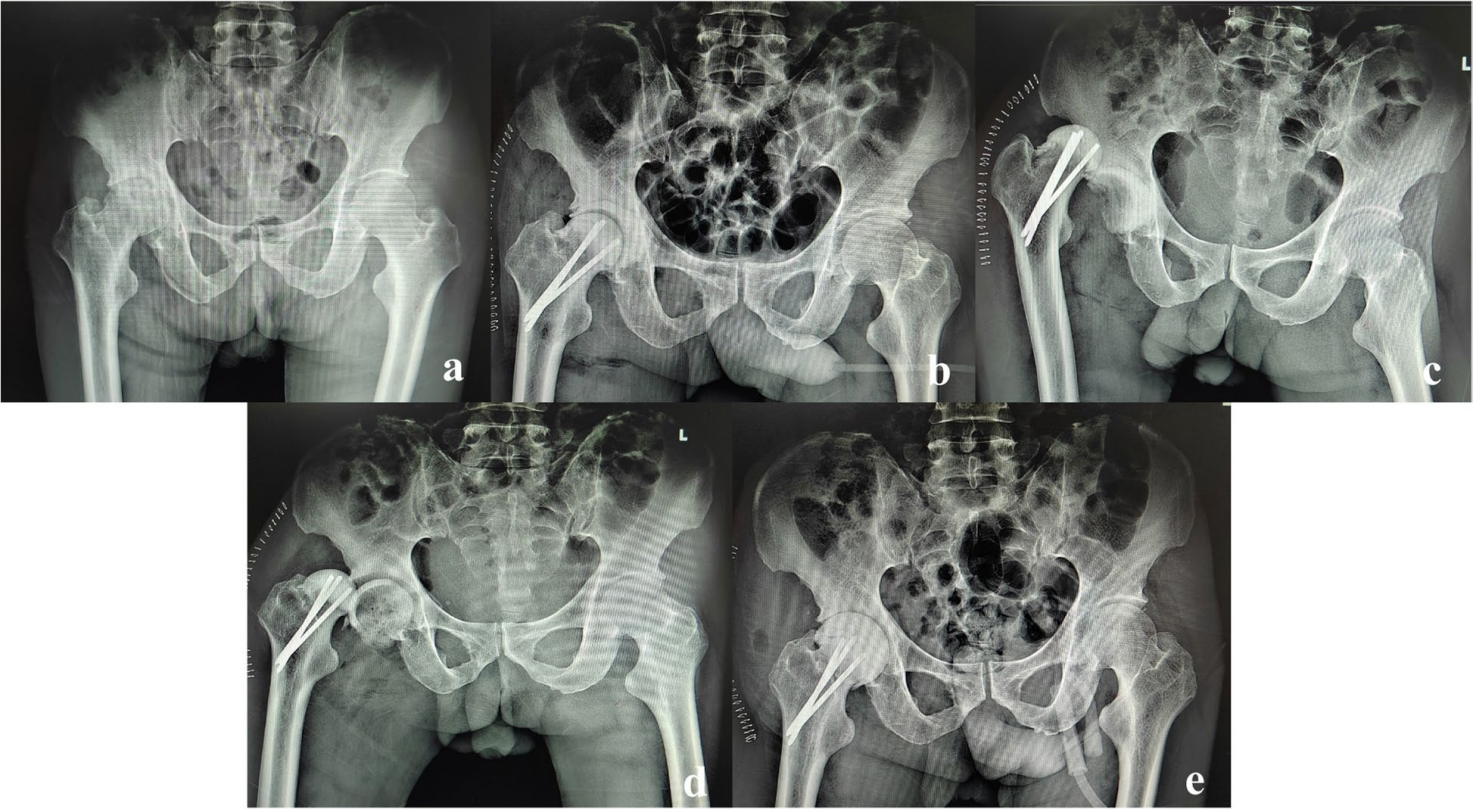
One case of antibiotic-load temporary spacer migration. A 73- year-old male patient was diagnosed with right hip purulent infection and underwent debridement and antibiotic-loaded bone cement temporary spacers. PD occurred 3 days after the operation, and the Allis maneuver was applied for reduction. Spacer migration was confirmed from the AP view of the pelvic X-ray film, and open reduction with spacer exchange was performed. **a**. Preoperative X-ray film of the pelvis. **b**. AP view X-ray film of the pelvis immediately after the operation. **c**. AP view X-ray film of the pelvis, showing PD occurred. **d**. AP view X-ray film of the pelvis immediately after closed reduction with the Allis maneuver, showing spacer migration. **e**. AP view X-ray film of the pelvis immediately after open reduction and spacer exchange operation

The reduction complications included 2 cases of periprosthetic fracture (Figs. 3 and 4) and 1 antibiotic-load temporary spacer migration (Fig. 5), which all occurred in the Allis group. The frequency of complications did not differ significantly between the two groups (*P* = 0.085).

The satisfaction results showed that the mean doctor and patient SAPS scores in the MLP group were 84.00 points and 76.97 points, respectively, which were significantly higher than those in the Allis group (72.12, *P* = 0.008 and 63.28 points, *P* = 0.001).

The safety assessment results showed that four adverse events were reported in the Allis group: (1) one doctor fell from the patient bed with soft tissue injury; (2) two doctors were diagnosed with lumbar muscle strain, and one was diagnosed with wrist deltoid cartilage complex injury. No adverse events were found in the MLP group.

## Discussion

Although THA is one of the most successful surgical procedures in the orthopedics field, postoperative complications cannot be ignored. According to the literature, the second most common complication after THA is dislocation following aseptic loosening [14, 20]. Gillinov et al. [21] followed up 155,185 primary THAs and found that the dislocation rate within 2 year was 2.3%. Even for dual mobility implants, a novel design to increase hip stability, the dislocation rate was still not low, affecting up to 0.9% to 3.0% of primary and revision patients [22]. For revision, the healthcare Cost and Utilization project nationwide inpatient sample database with 51,345 revision THA included revealed that the most common causes of revision were dislocation (22.5%), followed by mechanical loosening (19.7%) and infection (14.8%) [23]. The Dutch Arthroplasty Registry included 30,830 patients with hemiarthroplasty and THA for hip fractures, they found that joint dislocations, periprosthetic fractures, and infections were the common reasons for revision, with joint dislocations being the most common reason for revision (41%) [24]. Volpin et al. [25] systematically reviewed the literature and reported clinical outcomes and survival rates of contemporary revision acetabular arthroplasty and found that dislocation was the most common complication after acetabular reconstruction.

PD is the most common type of dislocation after THA, which requires immediate management to reduce pain, restore hip function, and avoid the risk of sciatic nerve palsy. Closed reduction is usually the first and most effective step in treatment. Achieving effective and safe closed reduction is a common problem faced by doctors. Bigelow first mentioned the maneuver of closed reduction in 1870 [10, 11]. Since then, various closed reduction methods have been introduced, and the outcomes have been uncertain [13]. At present, there are mainly two types of closed reduction maneuvers for PD: the supine reduction maneuver and the lateral reduction maneuver. The supine reduction method is represented by the Allis maneuver and has been modified by the Lefkowitz maneuver, Captain Morgan maneuver, East Baltimore Lift maneuver, Howard maneuver, etc. Taking the classic Allis maneuver as an example, there are some disadvantages in the reduction maneuver of the supine position [13, 14]: (1) the patient is supine with the affected limb upward. The doctor needs to exert more traction to resist gravity, and their backs are always straight to avoid having a back injury. (2) The doctor needs to enter the patient's bed or stretcher to apply traction. The risk of falling cannot be avoided even though doctors are advised to squat over the patient. The results of the doctor safety events of our study supported the above viewpoints: when doctors applied the Allis maneuver for reduction, one fell out of bed when pulling the leg and was diagnosed with soft tissue injury; two had low back pain and were diagnosed with psoas muscle strain and treated with NSAID drugs for 4 weeks; and one was diagnosed with wrist deltoid cartilage complex injury and wore a brace for immobilization without surgical treatment. The occurrence of the above safety events indicates that the Allis maneuver has certain doctor safety problems during the process of reduction. Waddell et al. [13], in their assessment of hip joint reduction operation techniques and doctors' safety, stated that the use of Allis maneuver in reduction would cause significant strength and tension in the back of doctors, and there was a risk of psoas muscle damage. In the Allis group of this study, the reduction doctor presented with lumbar muscle strain. Meanwhile, the satisfaction results than those in the MLP group, which indicates that the Allis maneuver places an enormous physical burden on doctors and patients due to the forceful and sustained traction.

The lateral reduction maneuver was first proposed by Skoff et al. in 1986 [15]. During the reduction process, the doctor stands beside the patient's bed and reduces the risk of falling. However, the doctor using this maneuver still needs to provide great traction, which might induce muscle strain of the lumbar muscle and arm. Subsequently, Dahners et al. [15] improved the Skoff method by using a traction-countertraction technique to increase traction force and make it safer for the doctor. Our MLP maneuver originates from the Skoff maneuver with some modification and imitates the reduction maneuver when performing a THA operation with a posterolateral approach. This modified maneuver has obvious advantages compared with the Allis maneuver, including the following: (1) the patient is placed in a lateral position with the affected limb downward. The total traction force is reduced without resisting the gravity of the limb; (2) the doctor can use body weight to provide traction and avoid back injury; (3) the doctor does not need to enter the patient’s bed, and there is no risk of falling; and (4) no assistive tool is needed during the reduction process, such as a traction table or strap [15, 26]. The results of this study proved that the MLP maneuver improved the success rate of reduction, reduced the incidence of complications and adverse events, and improved patient satisfaction, which showed obvious advantages compared with the Allis maneuver.

Some limitations were found in our present study. First, the sample size was not large, and more patients need to be enrolled to make the results more reliable. However, most previous studies only introduced the details of a modified reduction maneuver and seldom reported clinical studies to compare the differences between two reduction maneuvers, such as ours. Second, our study was a retrospective study. Our next step might be to perform a randomized controlled study, which can improve the comparability of the two maneuvers and eliminate confounding factors. Third, the reductions were performed by different doctors. Differences in reduction technique and traction force provided among doctors were the potential confounding factors between groups and might influence the final results. The fourth, the doctor's back pain was not only related to the way of reduction, but also related to the doctor's experience, the patient's position and the degree of patient’s cooperation. This can affect our judgment of the outcome of a safe incident.

## Conclusions

Our study demonstrated that for posterior dislocation after THA, the MLP reduction maneuver can effectively increase the reduction success rate, satisfaction scores, and doctor safety without increasing the risk of complications compared with the traditional Allis supine reduction maneuver. We recommend routinely using the MLP reduction maneuver instead of the Allis maneuver in THA patients with PD.

## Data Availability

The datasets used and/or analyzed during the current study are available from the corresponding author on reasonable request.

## Abbreviations

PD: Posterior dislocation
THA: Total hip arthroplasty
MLP: Modified lateral position
HHS: Harris hip score
ED: Emergency department
SAPS: Self-Administered patient satisfaction scale;
ONFH: Osteonecrosis of the femoral head
OA: Osteoarthritis
RA: Rheumatoid arthritis
DDH: Developmental dysplasia of the hip
COPD: Chronic obstructive pulmonary disease
